# The effect of transformational leadership on nurses’ turnover intention: the mediating effects of person-organization fit and career calling

**DOI:** 10.3389/fpsyg.2026.1880075

**Published:** 2026-10-05

**Authors:** Kailin Yin, Manman Shi, Lin Ji, Jie Sun, Binbin Zhang

**Affiliations:** 1 School of Nursing, Shandong Second Medical University, Weifang, China; 2 School of Nursing and Rehabilitation, Cheeloo College of Medicine, Shandong University, Jinan, China; 3 Department of General Surgery, Qilu Hospital of Shandong University, Jinan, China; 4 Department of Nursing, Qilu Hospital of Shandong University Dezhou Hospital, Dezhou, China

**Keywords:** career calling, nurses, person-organization fit, transformational leadership, turnover intention

## Abstract

**Aims:**

This study aims to explore the relationship between transformational leadership, person-organization fit, career calling, and turnover intention among nurses. Additionally, it investigates whether person-organization fit and career calling serve as independent and chain mediators in the link between transformational leadership and turnover intention.

**Methods:**

The researchers selected a convenience sample of 1,342 nurses from four Grade A-III hospitals in Shandong Province between October and November 2025. They collected data using several instruments: the General Population Questionnaire, the Transformational Leadership Questionnaire, the Person-Organization Fit Scale, the Career Calling Scale, and the Intent to Leave Scale. Descriptive statistics, correlation analysis, and regression analysis were conducted using SPSS 26.0. To examine the chained mediation effect, they applied Model 6 in PROCESS 3.5 and estimated the 95% confidence interval using the Bootstrap method with 5,000 repeated samples.

**Results:**

The results demonstrated that transformational leadership had a positive correlation with both person-organization fit and career calling, and a negative correlation with turnover intention. Mediation analysis revealed that over half of the protective effect of leadership on turnover intention was indirect, mediated through three pathways: person-organization fit alone, career calling alone, and their sequential chain. All indirect effects were statistically significant.

**Conclusion:**

These findings show that transformational leadership reduces turnover intention through various complementary psychological mechanisms. This highlights the importance of relational fit and intrinsic motivation in understanding leadership effectiveness. The sequential mediation pathway reveals how organizational and personal factors interact to influence nurses’ career decisions, offering a more nuanced theoretical framework for retention research.

## 1 Introduction

The global healthcare sector is grappling with a severe shortage of healthcare workers (Huo et al., 2025; Shin et al., 2024). High turnover rates are a major factor in the nursing shortage, with annual rates ranging from 15.1 to 44.3% in various countries (North et al., 2013; O'Brien-Pallas et al., 2010; Roche et al., 2015). A nationwide study in China found that 49.58% of clinical nurses wanted to leave their jobs (Wang et al., 2022). This ongoing loss of skilled nurses places a heavy financial burden on healthcare institutions (Alreshidi et al., 2021; Park et al., 2019). Beyond economic costs, high nurse turnover negatively impacts patient care; understaffed and unstable nursing teams compromise care quality and safety (Lee and De Gagne, 2022). Additionally, the departure of experienced nurses results in a loss of institutional knowledge and mentoring, increasing pressure on remaining staff. This shortage raises workloads, causing stress and burnout, which further fuels resignations (Rodríguez-García et al., 2024; Yang et al., 2017). Addressing nurse turnover is thus a critical priority in nursing management.

Among the various factors affecting nurse turnover, the leadership style of nursing managers emerges as a crucial determinant of nurses’ decisions to leave their positions (Magbity et al., 2020). Transformational leadership, in particular, is recognized as an effective management approach that encourages nursing staff to remain with an organization over the long term (Choi et al., 2016; Xie et al., 2020). While transformational leadership correlates with reduced turnover intentions among nurses, the mechanisms driving this effect warrant further investigation. To shed light on these mechanisms, this study introduces two pivotal psychological constructs: person-organization fit and career calling. Transformational leadership, person-organization fit, and career calling are key factors influencing nurses’turnover intentions. If nursing managers can comprehensively consider the interactions among these factors, they can implement effective measures to reduce nurse turnover rates. However, few studies have thoroughly explored the complex relationships among these variables. Therefore, the main research question guiding this study is: How does transformational leadership influence nurses’ turnover intention, and do person-organization fit and career calling serve as independent and chain mediators in this relationship?

## 2 Background

Social exchange theory provides a solid framework for understanding the relationship between leadership behavior and employee attitudes (Blau, 1964). This theory posits that when leaders offer valuable resources, employees feel obliged to reciprocate with positive attitudes and behaviors. Transformational leadership, in this context, is a crucial social exchange resource that affects nurses’ psychological states and, in turn, their intention to leave. Person-organization fit and career calling, which evaluate the deep connection between individuals, organizations, and their professions, may act as key mediators in this process. However, few studies have systematically examined how these psychological states mediate the relationship between transformational leadership and nurses’ intention to leave. This study, grounded in social exchange theory, investigates the links among transformational leadership, person-organization fit, career calling, and turnover intention in a sample of Chinese nurses.

### 2.1 Transformational leadership and turnover intention

Transformational leadership, as defined by Bass (1985), inspires and motivates subordinates to prioritize organizational goals over personal interests. By leading through example, offering a compelling vision, encouraging innovative thinking, and addressing individual developmental needs, transformational leaders can enhance employees’ psychological wellbeing and job performance while reducing turnover (Xiong et al., 2022). Research consistently shows a significant negative correlation between transformational leadership and nurse turnover (AbuAlRub and Nasrallah, 2017; Lavoie-Tremblay et al., 2016; Lyu et al., 2022; Magbity et al., 2020). This relationship is largely due to the leadership style’s capacity to improve nurses’ work experiences and alleviate stress and burnout. While other factors may mediate its impact (Brewer et al., 2016), transformational leadership is widely recognized for reducing nurses’ intentions to leave. Consequently, substantial evidence suggests a strong link between transformational leadership and nurses’ intention to stay in their roles.

### 2.2 Transformational leadership, person-organization fit, and turnover intention

Person-organization fit refers to the harmonious integration between an individual and an organization, highlighting the alignment, complementarity, and compatibility in culture, values, goals, and skills (Ding et al., 2010). Transformational leadership, person-organization fit, and turnover intention are closely interconnected concepts.

Transformational leadership is a key factor in achieving person-organization fit. Studies show it significantly and positively influences nurses’ alignment with their organizations (Liu et al., 2023). According to social exchange theory, transformational leaders align values, supply–demand, and competencies between nurses and the organization by establishing shared ideals, providing developmental resources, and granting autonomy (Han and Zhuang, 2020; Robbins and Davidhizar, 2020). Subordinates view this leadership style as a sign of trust and support, facilitating their adaptation. As a result, nurses become more proactive in integrating into the organization, thereby enhancing overall fit.

A high level of person-organization fit significantly influences employees’ intentions to leave, as supported by previous studies (Peng et al., 2014; Redelinghuys, 2023). When nurses perceive a strong alignment between their personal values and the hospital’s organizational culture, their work engagement and organizational commitment increase. This alignment fosters a positive psychological state, which directly reduces turnover intentions. Nurses become more committed to their work and develop a stronger sense of belonging to the organization. Based on these findings, we propose that transformational leadership may influence nurses’ turnover intentions through the mediating effect of person-organization fit.

### 2.3 Transformational leadership, career calling, and turnover intention

A sense of career calling integrates personal interests, values, and passions into one’s career, transcending mere employment. It represents a deep connection between an individual and their work, inspiring intrinsic motivation and providing meaning and purpose (Dobrow et al., 2023). Transformational leadership can effectively cultivate this sense of calling among nurses. By motivating, sharing a vision, and offering personalized support, transformational leaders enhance nurses’ alignment with their authentic selves. This alignment enables nurses to find profound meaning and value in their work, internalizing their profession as a true calling (Pan, 2025). A strong sense of career calling instills deep responsibility and meaning in nurses’ work. This intrinsic drive fuels motivation and enthusiasm, encouraging proactive coping strategies in the face of challenges and a commitment to providing high-quality patient care. Consequently, nurses experience a heightened sense of self-worth. Because their work holds such profound meaning, they are more likely to remain in their positions despite difficulties, leading to lower turnover intentions (Lin et al., 2025). In the nursing profession, career calling serves as a powerful internal motivator, potentially offering greater predictive power for turnover intentions than traditional variables. However, few studies have incorporated this concept into models of leadership influence. Therefore, we propose the hypothesis that career calling mediates the relationship between transformational leadership and turnover intention.

### 2.4 Person-organization fit and career calling

Person-organization fit and career calling may mediate the link between transformational leadership and turnover intention. Notably, these variables exhibit a sequential relationship, with person-organization fit as a key precursor influencing career calling (Xu et al., 2023). Person-organization fit strengthens the psychological contract between employees and the organization, fostering intrinsic identification and a sense of meaning in work. This intrinsic career calling, nurtured by person-organization fit, reduces turnover intentions among nurses by enhancing work resilience and intrinsic satisfaction. Previous research has often focused on single mediating variables, overlooking the possibility that transformational leadership might influence nurses’ turnover intentions through multiple pathways, including person-organization fit and a personal sense of mission. Therefore, this study aims to uncover a comprehensive chain-mediation mechanism. Specifically, we propose that transformational leadership boosts person-organization fit, which then enhances nurses’ sense of career calling, ultimately reducing their turnover intentions.

## 3 Purpose of this study

This study examines the mechanisms linking transformational leadership, person-organization fit, career calling, and turnover intention among nurses in China. Based on existing literature and theoretical frameworks, we propose four research hypotheses:

### 3.1 Direct hypothesis

*H1*: Transformational leadership has a significant negative direct effect on nurses’ intention to leave.

### 3.2 Indirect hypotheses

*H2*: Transformational leadership indirectly influences nurses’ intention to leave through the mediating effect of person-organization fit.

*H3*: Transformational leadership indirectly influences nurses’ intention to leave through the mediating effect of career calling.

*H4*: Transformational leadership indirectly influences nurses’ intention to leave through the chained mediating effects of person-organization fit and career calling.

## 4 Materials and methods

### 4.1 Study design

This study employed a descriptive cross-sectional survey design, following the STROBE guidelines to improve the reporting quality of observational epidemiological studies (von Elm et al., 2008).

### 4.2 Study setting and sampling

The study recruited participants through convenience sampling from nurses at four Grade A tertiary hospitals in Shandong Province between October and November 2025. According to Kendall’s sample size estimation method (Xin et al., 2024), a descriptive study needs a sample size 5–10 times the number of variables. The demographic questionnaire in this study included 6 sociodemographic variables and 4 scales, totaling 59 variables. Preliminary calculations indicated a sample size of 295–590 participants. Considering a 20% dropout rate, the required sample size ranged from 369 to 738 participants. Ultimately, 1,342 participants were included in the study.

### 4.3 Inclusion and exclusion criteria

The inclusion criteria were: (1) possessing a valid nursing license, (2) having at least 1 year of work experience, and (3) providing informed consent and agreeing to voluntary participation in the study. The exclusion criteria included: (1) nurses currently on rotation or in training, (2) nurses absent for training or on leave for a minimum of 1 month during the study, and (3) nurses who had recently experienced a major traumatic event.

### 4.4 Instruments

#### 4.4.1 Nurse characteristics

The researchers developed a nurse profile questionnaire comprising questions on age, gender, marital status, educational background, professional title, job level, and years of service.

#### 4.4.2 Turnover intention questionnaire

Michaels and Spector (1982) created a scale to assess employees’ intention to leave, consisting of three dimensions and six items. Participants respond on a four-point rating scale (1 = “never” to 4 = “often”). The total score ranges from 6 to 24, where higher scores indicate a stronger intention to leave. This study used the Chinese version of the scale (Lee, 2003). Because each sub-dimension contains only two items, a three-factor CFA would be just-identified; therefore, a one-factor CFA was carried out instead. The model showed acceptable fit, yielding *χ*^2^/df = 2.62, CFI = 0.996, TLI = 0.993, SRMR = 0.011, and RMSEA = 0.035. All six standardized factor loadings were statistically significant (*p* < 0.001), ranging from 0.691 to 0.777. Internal consistency was satisfactory, with a Cronbach’s α of 0.882.

#### 4.4.3 Transformational leadership questionnaire

This study utilized a peer-rated questionnaire, developed by Lee Chaoping, to evaluate employees’ perceptions of managers’ transformational leadership behaviors within the Chinese cultural context (Lee and Shi, 2005). The questionnaire comprises four dimensions: moral exemplification, visionary inspiration, charisma, and personalized concern, totaling 26 items. Each item is rated on a 5-point Likert scale, from 1 (strongly disagree) to 5 (strongly agree). A higher total score indicates that nurses perceive their managers as demonstrating stronger transformational leadership behaviors. We subjected the 26 items to a four-factor CFA to verify this structure. Fit indices suggested satisfactory model fit: *χ*^2^/df = 1.87, CFI = 0.988, TLI = 0.986, SRMR = 0.019, and RMSEA = 0.025. Standardized loadings for all items were significant (*p* < 0.001) and fell between 0.684 and 0.756. The scale demonstrated strong internal consistency, with a Cronbach’s α of 0.963.

#### 4.4.4 Person-organization fit scale

The Person-Organization Fit Scale, developed by Cable and DeRue (2002), comprises three dimensions: value fit, needs-organization supply fit, and organizational capability fit. It includes nine items rated on a 5-point Likert scale, ranging from 1 (strongly disagree) to 5 (strongly agree). Higher scores suggest a better fit between the individual and the organization. Xi (2012) adapted this scale for use in China. This study used the revised Chinese version. CFA was used to corroborate the hypothesized three-factor measurement model. The analysis produced adequate fit statistics (*χ*^2^/df = 2.52, CFI = 0.994, TLI = 0.991, SRMR = 0.014, RMSEA = 0.034), and every standardized item loading reached significance (*p* < 0.001), varying from 0.632 to 0.761. Reliability analysis yielded a Cronbach’s α of 0.907.

#### 4.4.5 Calling scale

The Career Calling Scale, initially developed by Dobrow and Tosti-Kharas (2011) and subsequently translated into Chinese by Pei and Zhao (2015), is a unidimensional tool consisting of 12 items. Scores range from 1 to 5, with higher scores indicating a stronger subjective sense of occupational value and increased intrinsic motivation at work. Unidimensionality was assessed with a single-factor CFA, which returned favorable fit: *χ*^2^/df = 1.71, CFI = 0.995, TLI = 0.994, SRMR = 0.014, and RMSEA = 0.023. The 12 standardized factor loadings were all significant (*p* < 0.001), spanning 0.690–0.731. The scale also showed excellent internal consistency, reflected by a Cronbach’s α of 0.924.

### 4.5 Data collection

Data collection utilized the electronic survey platform Wenjuanxing. Prior to the survey launch, we created digital posters containing the online survey link. With the nursing departments’ approval at each hospital, these posters were shared with nurses via the WeChat social media platform. Each questionnaire included detailed instructions explaining key concepts, the survey’s objectives, and its importance. We guaranteed participants strict confidentiality of their responses and required signed informed consent forms. Nurses completed the questionnaire independently and anonymously. After submission, two researchers verified identity information and related data in the electronic survey system to ensure accuracy, excluding any questionnaires with incomplete or invalid responses.

### 4.6 Data statistical analysis

In this study, we used SPSS (Version 26.0) for data analysis, setting a significance level of 0.05 for the research variables. Descriptive statistics assessed sample characteristics and measurement instrument scores. Univariate analyses were performed to compare turnover intention scores across different demographic groups. All variables were treated as observed composite scores (i.e., the arithmetic mean of item scores for each scale) and entered as single indicators in the regression models. Prior to hypothesis testing, confirmatory factor analyses (CFA) were conducted for each measurement instrument using R software with the lavaan package to verify the hypothesized factor structures, and standardized factor loadings together with model fit indices (*χ*^2^/df, CFI, TLI, SRMR, and RMSEA) were examined. Reliability and convergent validity were evaluated using Cronbach’s α coefficients, composite reliability (CR), and average variance extracted (AVE), while discriminant validity was assessed using the heterotrait–monotrait ratio of correlations (HTMT). In addition, the variance inflation factor (VIF) was calculated to diagnose multicollinearity among the predictor variables, with values below 5 considered indicative of no serious multicollinearity. Pearson’s correlation analysis examined relationships among variables. To examine the chained mediation effect, we employed Model 6 of the PROCESS macro (Version 3.5) for SPSS (Hayes, 2018). In this model, transformational leadership was the independent variable, turnover intention was the dependent variable, person-organization fit and career calling were the serial mediators, and only demographic variables that showed statistical significance in the univariate analyses were included as covariates. The significance of direct and indirect effects was assessed using the bias-corrected Bootstrap method with 5,000 resamples to estimate 95% confidence intervals.

### 4.7 Ethical approval

The Ethics Committee of Qilu Hospital of Shandong University Dezhou Hospital, approved this study (Ethics Approval No.: 2025115). All participants provided informed consent and participated voluntarily.

## 5 Results

### 5.1 Assessment of common method bias

To evaluate potential common-method bias from the self-report questionnaires in this study, we used Harman’s single-factor test for statistical analysis (Podsakoff et al., 2003). We included all 53 measurement items in the exploratory factor analysis. The analysis identified five factors with eigenvalues greater than 1. The first factor explained 35.31% of the variance, which is below the critical threshold of 40%. This result indicates no significant common-method bias, enabling us to proceed with the subsequent mediation analyses.

### 5.2 The demographic characteristics of the participants

This study included 1,342 participants, with their sociodemographic characteristics detailed in Table 1. The analysis revealed a predominantly female population, comprising 1,233 participants, or 91.9%. The average age mainly fell within the 31–40 range, representing 698 participants, or 52.0%. Most participants were married, totaling 1,024 individuals, or 76.3%. In terms of education, 1,191 participants, or 88.7%, held a bachelor’s degree. Senior nurses constituted the largest group by professional title, with 644 participants, or 48.0%. Regarding job level, nearly half, 665 individuals or 49.6%, were at the N3 level.

**Table 1:** Social–demographic characteristics of participants (*N* = 1,342).

Variables	*N* (%)	Turnover intention	*t*/*F*	*p*
Age (years)				
18–25	188 (14.0)	11.78 ± 4.27	5.466	<0.01
26–30	201 (15.0)	12.33 ± 3.91		
31–35	290 (21.6)	12.66 ± 4.09		
36–40	408 (30.4)	12.68 ± 3.96		
>40	255 (19.0)	13.55 ± 4.37		
Gender				
Male	109 (8.1)	12.32 ± 4.14	−0.892	>0.05
Female	1,233 (91.9)	12.69 ± 4.13		
Marital status				
Single	280 (20.9)	12.27 ± 3.63	10.521	<0.01
Married	1,024 (76.3)	12.86 ± 4.18		
Divorced and widowed	38 (2.8)	10.00 ± 5.16		
Educational attainment				
College diploma	87 (6.5)	11.44 ± 4.47	22.499	<0.01
Bachelor degree	1,191 (88.7)	12.91 ± 4.02		
Master’ degree	64 (4.8)	9.75 ± 4.26		
Professional title				
Registered nursing	182 (13.6)	12.87 ± 3.86	11.615	<0.01
Senior nurse	359 (26.8)	12.16 ± 3.90		
Nursing-in-charge	644 (48.0)	13.21 ± 4.22		
Associate chief nurse and above	157 (11.7)	11.31 ± 4.17		
Position level				
*N*1	325 (24.2)	13.29 ± 4.31	10.897	<0.01
*N*2	186 (13.9)	12.84 ± 4.00		
*N*3	665 (49.6)	12.69 ± 3.83		
*N*4	166 (12.4)	11.09 ± 4.68		

### 5.3 Correlation analysis of major study variable

The Pearson correlation analysis revealed that transformational leadership positively correlated with person-organization fit (*r* = 0.409, *p* < 0.01) and career calling (*r* = 0.510, *p* < 0.01). In contrast, it negatively correlated with nurses’ intention to leave (*r* = −0.419, *p* < 0.01). Furthermore, person-organization fit showed a positive correlation with career calling (*r* = 0.485, *p* < 0.01) and a negative correlation with nurses’ intention to leave (*r* = −0.447, *p* < 0.01). Career calling also had a negative correlation with nurses’ intention to leave (r = −0.484, *p* < 0.01). For detailed data, see Table 2.

**Table 2:** Pearson’s correlation analyses between transformational leadership, person-organization fit, career calling, and nurses’ retention intention (*N* = 1,342).

Variables	*M*	SD	1	2	3	4
1. Transformational leadership	81.71	18.73	1			
2. Person-organization fit	27.86	6.47	0.409^**^	1		
3. Career calling	35.94	8.49	0.510^**^	0.485^**^	1	
4. Nurses’ turnover intention	12.66	4.13	−0.419^**^	−0.447^**^	−0.484^**^	1

*M*, mean; SD, standard deviation.

^∗∗^*p* < 0.01.

### 5.4 Construct validity and reliability

In addition to the confirmatory factor analysis (CFA) results reported in the Measures section, which demonstrated good model fit for each construct and provided initial evidence of construct validity, we further examined the psychometric properties of the scales using Average Variance Extracted (AVE), Composite Reliability (CR), and the Heterotrait–Monotrait ratio (HTMT). All constructs exhibited sufficient internal consistency (CR ≥ 0.70) and acceptable convergent validity (AVE ≥ 0.50), as shown in Table 3. Furthermore, all construct pairs had HTMT values below 0.85, indicating discriminant validity. Taken together, the CFA evidence and these reliability and validity indices confirm that the study’s measures are psychometrically sound and appropriate for subsequent mediation analyses. The variance inflation factor (VIF) values of all predictors were below 5, indicating that multicollinearity was not a concern.

**Table 3:** Construct validity and reliability.

Construct	AVE	CR	HTMT (vs other constructs)
Turnover Intention (TI)	0.557	0.883	0.452 (vs TL) / 0.499 (vs POF) / 0.535 (vs CC)
Transformational Leadership (TL)	0.518	0.965	0.452 (vs TI) / 0.436 (vs POF) / 0.540 (vs CC)
Person–Organization Fit (POF)	0.519	0.906	0.499 (vs TI) / 0.436 (vs TL) / 0.527 (vs CC)
Calling (CC)	0.505	0.924	0.535 (vs TI) / 0.540 (vs TL) / 0.527 (vs POF)

AVE, Average Variance Extracted; CR, Composite Reliability; HTMT, Heterotrait–Monotrait ratio.

### 5.5 The chain mediating analysis

Table 4 shows the regression analysis results. Transformational leadership significantly negatively predicted nurses’ intention to leave (*β* = −0.399, *p* < 0.001), supporting Hypothesis H1. It also positively predicted person-organization fit (*β* = 0.389, *p* < 0.001) and career calling (*β* = 0.357, *p* < 0.001). Person-organization fit positively predicted career calling (*β* = 0.311, *p* < 0.001) and negatively predicted turnover intention (*β* = −0.231, *p* < 0.001). Career calling significantly negatively predicted turnover intention (*β* = −0.262, *p* < 0.001). Even after controlling for person-organization fit and career calling, transformational leadership’s direct effect on turnover intention remained significant (*β* = −0.184, *p* < 0.001), indicating a partial mediating effect.

**Table 4:** Regression analysis of the relationship between transformational leadership and turnover intention.

Regression equation		Fitting index		Significance	
Outcomes	Predictors	*R* ^2^	*F*	*β*	*t*
Turnover intention		0.204	56.943		
	TL			−0.399	−16.062^***^
	Covariates^1^				
Person-organization fit		0.185	50.512		
	TL			0.389	15.473^***^
	Covariates^1^				
Career calling		0.372	113.026		
	TL			0.357	14.903^***^
	POF			0.311	12.953^***^
	Covariates^1^				
Turnover intention		0.326	80.633		
	TL			−0.184	−6.867^***^
	POF			−0.231	−8.722^***^
	CC			−0.262	−9.225^***^
	Covariates^1^				

*N* = 1,342. TL, Transformational Leadership; POF, Person-Organization Fit; CC, Career Calling.

1 Covariates include age, marital status, educational attainment, Professional title, position level.

^***^p < 0.001.

Table 5 and Figure 1 illustrate how person-organization fit and career calling mediate the relationship between transformational leadership and nurses’ intention to leave. The total effect of transformational leadership on this intention was −0.088, comprising a direct effect of −0.041 and a total indirect effect of −0.047. This mediation included three indirect paths. Path 1 (Transformational Leadership → Person-Organization Fit → Intent to Leave) had an effect size of −0.020, with a 95% confidence interval of (−0.025, −0.015), contributing 22.50% to the total effect. Path 2 (Transformational Leadership → Career Calling → Intent to Leave) showed an effect size of −0.021 and a 95% confidence interval of (−0.026, −0.015), accounting for 23.41% of the total effect. Path 3 (Transformational Leadership → Person-Organization Fit → Career Calling → Intent to Leave) had an effect size of −0.007, with a 95% confidence interval of (−0.009, −0.005), representing 7.95% of the total effect. All three indirect effects were statistically significant, as their 95% confidence intervals did not include zero, thereby supporting Hypotheses 2, 3, and 4.

**Table 5:** Total, direct, and indirect effects of the mediation model.

Effect	Product of coefficients		Bootstrapping 95% CI	
	Point estimate	Boot SE	Lower	Upper
Total effect of TL on TI	−0.088	0.006	−0.099	−0.077
Direct effect of TL on TI	−0.041	0.006	−0.052	−0.029
Total indirect effect of TL on TI	−0.047	0.004	−0.055	−0.040
Indirect1: TL → POF → TI	−0.020	0.003	−0.025	−0.015
Indirect2: TL → CC → TI	−0.021	0.003	−0.026	−0.015
Indirect3: TL → POF → CC → TI	−0.007	0.001	−0.009	−0.005

TL, Transformational Leadership; POF, Person-Organization Fit; CC, Career Calling; TI, Turnover Intention.

**Figure 1:**
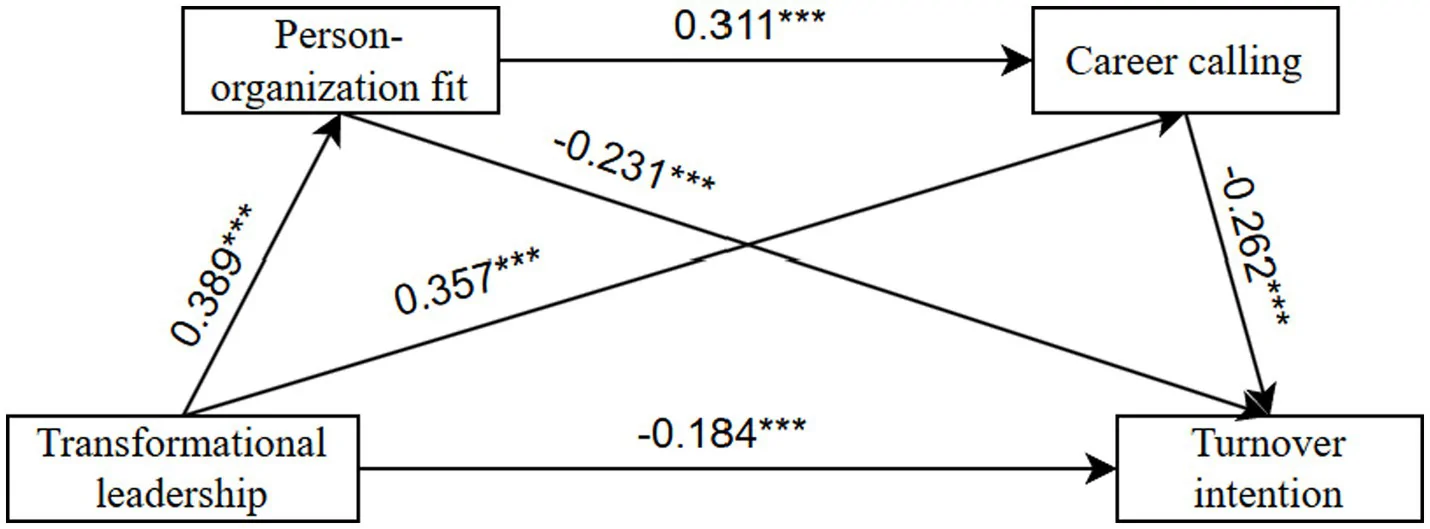
Standardized path coefficients for the chain mediation model. ***p < 0.001.

## 6 Discussion

This study, grounded in social exchange theory, developed and tested a chained mediation model to explore how transformational leadership affects nurses’ turnover intentions. Analyzing data from 1,342 nurses in four tertiary hospitals in Shandong Province, China, we discovered that transformational leadership significantly decreased turnover intention through four pathways: a direct effect accounting for 46.6% of the total effect, an indirect effect via person-organization fit at 22.5%, an indirect effect through career calling at 23.4%, and a chain-mediated effect via person-organization fit followed by career calling at 8.0%. All hypotheses were supported, with 95% confidence intervals for indirect effects excluding zero. These results suggest that transformational leadership’s impact on nurse retention is complex, involving both immediate psychological benefits and sequential organizational-personal mechanisms. Notably, the chain mediation pathway illustrates how transformational leadership enhances nurses’ perceived organizational alignment, which then fosters a stronger professional calling, ultimately reducing their intention to leave.

### 6.1 Direct effect of transformational leadership on turnover intention (H1)

This study confirmed Hypothesis 1 by showing a significant negative direct effect of transformational leadership on nurses’ turnover intention (*β* = −0.184, *p* < 0.001), even after accounting for two mediators. This aligns with previous research highlighting transformational leadership’s protective role against employee turnover in various occupational settings (AbuAlRub and Nasrallah, 2017; Lavoie-Tremblay et al., 2016; Lyu et al., 2022; Magbity et al., 2020). Notably, the direct effect (*β* = −0.041) accounted for about 46.6% of the total effect (*β* = −0.088), indicating that much of transformational leadership’s impact on nurse retention is not mediated by person-organization fit and career calling. This significant direct pathway may reflect the immediate psychological benefits provided by transformational leaders, such as emotional support, role clarity, and reduced job stress. These benefits directly enhance nurses’ job satisfaction and organizational attachment without necessarily changing their perceived organizational fit or professional calling (Brewer et al., 2016).

From the perspective of social exchange theory (Blau, 1964; Homans, 1958), the direct effect is seen as a relational exchange. When nurse managers consistently exhibit idealized influence, inspirational motivation, intellectual stimulation, and individualized consideration, nurses view these behaviors as valuable socioemotional resources. This perception fosters a sense of obligation to reciprocate with loyalty and retention. Unlike transactional and short-term economic exchanges, this socioemotional exchange cultivates a high-quality leader-member relationship. This relationship independently buffers against turnover intention, regardless of nurses’ cognitive assessments of organizational fit or professional purpose.

This direct effect may be more pronounced in the Chinese healthcare setting. In China’s high power distance culture, nurses heavily depend on leadership support, with supervisors acting as key organizational representatives (Hofstede, 2001). Furthermore, ongoing healthcare reforms have heightened work pressures, making transformational leadership an essential stress-buffering resource. This leadership style reduces turnover by influencing affective rather than cognitive pathways.

### 6.2 Person-organization fit as a mediator (H2)

This study confirmed Hypothesis 2, demonstrating that transformational leadership positively predicted person-organization fit (*β* = 0.389, *p* < 0.001), which, in turn, negatively predicted turnover intention (*β* = −0.231, *p* < 0.001). The indirect effect through this pathway was −0.020, accounting for 22.5% of the total effect. This finding aligns with previous research indicating that transformational leaders enhance employees’ organizational identification by aligning values and providing developmental resources (Hannah et al., 2016; Liu et al., 2023). From a social exchange perspective, transformational leadership acts as an organizational socialization mechanism. Leaders who articulate compelling visions and demonstrate value-congruent behaviors help nurses internalize organizational goals, fostering a sense of compatibility with the institution. This perceived fit reduces turnover intention by strengthening organizational commitment and perceived organizational support (Peng et al., 2014). Notably, person-organization fit and career calling showed similar mediation effects (22.5% vs. 23.4%), suggesting that both organizational-level and individual-level mechanisms contribute equally to transformational leadership’s protective effect. In the Chinese healthcare context, where nursing workforce stability is crucial amid ongoing reforms, enhancing person-organization fit represents a structural and cost-effective intervention target for hospital administrators.

### 6.3 Career calling as a mediator (H3)

This study confirmed Hypothesis 3. Transformational leadership positively predicted career calling (*β* = 0.357, *p* < 0.001), which in turn negatively predicted turnover intention (*β* = −0.262, *p* < 0.001). The indirect effect through this pathway was −0.021, accounting for 23.4% of the total effect, similar to the person-organization fit pathway. This finding extends prior research by showing that transformational leaders foster professional calling through self-concordance mechanisms. By providing intellectual stimulation and individualized consideration, leaders help nurses align their work with personal values and passions (Pan, 2025). Career calling reflects an individual’s intrinsic sense of meaning, passion, and mission toward a profession (Dik and Duffy, 2009; Dobrow and Tosti-Kharas, 2011). From a social exchange perspective, it represents an internalized return on investment. When leaders offer trust, autonomy, and growth opportunities, nurses transform these resources into a deeper vocational identity that transcends organizational boundaries. This intrinsic motivation makes nurses more resilient to job stress and less susceptible to turnover triggers (Zhu et al., 2023). Notably, the effect size of career calling nearly matched that of person-organization fit, suggesting that nurses’ retention decisions are influenced as much by professional purpose as by organizational alignment. In high-burnout settings such as Chinese tertiary hospitals, nurturing career calling offers a sustainable, low-cost retention strategy that operates independently of structural organizational changes.

### 6.4 Chain mediation effect (H4)

This study confirmed Hypothesis 4, showing that the chain-mediated pathway—transformational leadership → person-organization fit → career calling → turnover intention—was statistically significant (effect = −0.007, 95% CI [−0.009, −0.005]), accounting for 7.95% of the total effect. Although modest, this proportion reveals a sequential psychological mechanism. Transformational leadership initially enhances nurses’ perceived alignment with the organization, which then fosters a deeper sense of professional calling, ultimately reducing turnover intention (Xu et al., 2023). This finding extends social exchange theory by illustrating multi-stage resource internalization. Leaders provide socioemotional resources that nurses first interpret at the organizational level (fit), then transform into professional identity (calling), and finally reciprocate through retention. Unlike parallel mediation models that treat fit and calling as independent mechanisms, the chain mediation reveals their causal dependency: person-organization fit is essential for career calling to develop. Notably, the chain effect (7.95%) was smaller than either single mediation pathway (22.5 and 23.4%), indicating that fit and calling primarily operate as parallel rather than sequential mechanisms in this sample. Nonetheless, the chain pathway’s significance supports Xu et al.'s (2023) theoretical proposition that person-environment fit strengthens psychological contracts, fostering intrinsic work identification. For nursing managers, this suggests a staged intervention strategy: first, enhance organizational fit, then build upon this foundation to nurture career calling.

### 6.5 Theoretical contributions

This study builds on social exchange theory by showing that transformational leadership decreases turnover intention through several complementary pathways: a direct effect (46.6%), two single mediation pathways via person-organization fit (22.5%) and career calling (23.4%), and a chain mediation pathway through person-organization fit followed by career calling (7.95%). The similar effect sizes of fit and calling indicate that both organizational alignment and intrinsic motivation equally influence retention decisions. Furthermore, the chain mediation pathway confirms that person-organization fit acts as a precursor to career calling, highlighting how organizational socialization precedes the formation of professional identity.

### 6.6 Implications for policy and practice

Healthcare institutions should prioritize leadership development programs to cultivate transformational leadership behaviors among nurse managers. This study shows that transformational leadership is crucial in reducing turnover intention. Organizational policies should address structural factors influencing person-organization fit. These include transparent recruitment processes, clear role definitions, value-aligned performance evaluations, and opportunities for nurses to engage in decision-making. Nurse leaders should promote career calling among frontline staff through mentorship, meaningful work design, autonomy support, and recognition of professional contributions. These strategies, which enhance relational fit and intrinsic motivation, may effectively reduce turnover intention and support long-term workforce retention in nursing.

### 6.7 Limitations and future research

This study faced several limitations. First, its cross-sectional design prevented causal inferences. While mediation analysis with bootstrapping offered strong statistical evidence, longitudinal studies are necessary to establish temporal precedence. Second, the sample was limited to four tertiary hospitals in Shandong Province, which restricts the generalizability of findings to other regions or hospital tiers. Third, the use of self-reported questionnaires might introduce common method bias. Although Harman’s single-factor test showed no significant threat, future research should include objective turnover records or supervisor ratings. Fourth, the direct effect accounted for 46.6% of the total effect, indicating potential unmeasured mediators such as psychological safety, leader-member exchange quality, or work engagement. Future studies should investigate these pathways to develop a more comprehensive model. Lastly, experimental or intervention studies are needed to determine if transformational leadership training leads to the expected changes in fit, calling, and turnover intention.

## 7 Conclusion

Our study, grounded in social exchange theory, examined the interplay among leadership, organizations, and individuals. We found that person-organization fit and career calling significantly mediated the link between transformational leadership and turnover intention. Nursing managers should focus on effective leadership and fostering a fair, reciprocal organizational environment. This strategy enhances nurses’ alignment with the organization and strengthens their career calling, which ultimately reduces turnover intention.

## Data Availability

The raw data supporting the conclusions of this article will be made available by the authors, without undue reservation.
